# NI-1: a novel canine mastocytoma model for studying drug resistance and IgER-dependent mast cell activation

**DOI:** 10.1111/j.1398-9995.2012.02833.x

**Published:** 2012-05-15

**Authors:** E Hadzijusufovic, B Peter, H Herrmann, T Rülicke, S Cerny-Reiterer, K Schuch, L Kenner, T Thaiwong, V Yuzbasiyan-Gurkan, W F Pickl, M Willmann, P Valent

**Affiliations:** 1 Department for Companion Animals and Horses, Clinic for Internal Medicine and Infectious Diseases, University of Veterinary Medicine ViennaVienna, Austria; 2 Division of Hematology & Hemostaseology, Department of Internal Medicine I, Medical University of ViennaVienna, Austria; 3 Ludwig Boltzmann Cluster OncologyVienna, Austria; 4 Institute of Laboratory Animal Science, University of Veterinary Medicine ViennaVienna, Austria; 5 Institute of Immunology, Medical University of ViennaVienna, Austria; 6 Clinical Institute of Pathology, Medical University of ViennaVienna, Austria; 7 Ludwig Boltzmann Institute for Cancer ResearchVienna, Austria; 8 Comparative Medicine and Integrative Biology Program, Michigan State UniversityEast Lansing, MI, USA

**Keywords:** exon 8 mutation, Kit, mastocytoma, resistance, tyrosine kinase inhibitors

## Abstract

**Background:**

Advanced mast cell (MC) disorders are characterized by uncontrolled growth of neoplastic MC in various organs, mediator-related symptoms, and a poor prognosis. *Kit* mutations supposedly contribute to abnormal growth and drug resistance in these patients.

**Methods:**

We established a novel canine mastocytoma cell line, NI-1, from a patient suffering from MC leukemia.

**Results:**

NI-1 cells were found to form mastocytoma lesions in NOD/SCID IL-2Rgamma^null^ mice and to harbor several homozygous *Kit* mutations, including missense mutations at nucleotides 107(C→T) and 1187(A→G), a 12-bp duplication (nucleotide 1263), and a 12-bp deletion (nucleotide 1550). NI-1 cells expressed several MC differentiation antigens, including tryptase, Kit, and a functional IgE receptor. Compared to the C2 mastocytoma cell line harboring a *Kit* exon 11 mutation, NI-1 cells were found to be less responsive against the Kit tyrosine kinase inhibitors (TKI) masitinib and imatinib, but were even more sensitive against proliferation-inhibitory effects of the mammalian target of rapamycin (mTOR) blocker RAD001 and PI3-kinase/mTOR blocker NVP-BEZ235. The Kit-targeting multikinase inhibitors PKC412 and dasatinib were also found to override TKI resistance in NI-1 cells, and produced growth inhibition with reasonable IC_50_ values (<0.1 μM).

**Conclusion:**

NI-1 may serve as a useful tool to investigate IgE-dependent reactions and mechanisms of abnormal growth and drug resistance in neoplastic MC in advanced mastocytosis.

Mastocytosis is a heterogeneous group of neoplasms characterized by abnormal growth and accumulation of mast cells (MC) in one or more organ systems (1, 2). Cutaneous and systemic variants of the disease have been described (1). Systemic mastocytosis (SM) can show an indolent or an aggressive clinical course (1, 2). Mast cell leukemia (MCL) is the leukemic variant of SM (1, 2). In typical cases, MC account for ≥20% of all nucleated cells in bone marrow smears and ≥10% of peripheral blood leukocytes (1). Mast cell leukemia and aggressive SM (ASM) are characterized by rapid disease progression and a poor prognosis (2). In MCL, MC are particularly immature and show rapid proliferation in various organs with consecutive organ damage (1). In all disease variants, patients with mastocytosis suffer from mediator-related symptoms, especially when IgE-dependent reactions occur (1, 2).

A number of different conventional drugs and chemotherapy agents have been suggested for the treatment of patients with advanced SM; especially, interferon-alpha (IFNα) and cladribine (2CdA) were found to induce responses in patients with smoldering SM or slowly progressing ASM (3–5). However, in rapidly progressing ASM or MCL, the response against these agents is usually short-lived (3–5).

A number of previous and more recent data suggest that Kit tyrosine kinase inhibitors (TKI) suppress the growth of neoplastic MC (6–8). However, depending on the variant of SM and molecular defects, neoplastic MC may be resistant against TKI. Likewise, the *Kit* mutation D816V that is found in most patients with SM confers resistance against imatinib (6, 9). Some of the second-generation TKI like dasatinib or PKC412 have been reported to override drug resistance in Kit D816V-transformed cells (6–8). These agents are currently tested in clinical trials in advanced SM (10–12).

Recent data suggest that transforming *Kit* mutations are also found in canine mastocytomas (8, 13–15). These mutations are detected in *Kit* exons 8, 9, 11, 12, or 17 (13–15). None of these mutations confer resistance against imatinib or masitinib (16, 17). Therefore, both drugs have been considered for the treatment of canine mastocytomas (16–20). More recently, masitinib has received approval for the treatment of malignant mastocytomas in canines. However, although clinical responses are seen, they are usually short-lived and may be followed by a relapse (19).

The mechanisms of resistance of canine mastocytoma cells against masitinib remain at present unknown. Among others, one possibility might be that more malignant subclones bear or develop additional *Kit* mutations that confer resistance. One approach to study the mechanism(s) of resistance to masitinib is to establish novel cell line models. We have established a novel canine mastocytoma cell line designated NI-1. This cell line harbors multiple *Kit* mutations and a functional IgE receptor (IgER) and was found to respond differentially to various TKI.

## Materials and methods

### Reagents

The TKI bosutinib, dasatinib, imatinib, sorafenib, sunitinib, and nilotinib, the PI3 kinase/mammalian target of rapamycin (mTOR) blocker NVP-BEZ235, everolimus, the ErbB receptor inhibitors lapatinib, erlotinib, and gefitinib, the Aurora kinase inhibitor tozasertib, and the histone deacetylase (HDAC) inhibitor vorinostat were purchased from ChemieTek (Indianapolis, IN, USA), and masitinib and midostaurin from LC Laboratories (Woburn, MA, USA) (Table 1). Stock solutions were prepared by dissolving in dimethylsulfoxide (Merck, Darmstadt, Germany). RPMI 1640 medium and fetal calf serum (FCS) were purchased from PAA Laboratories (Pasching, Austria), Iscove's modified Dulbecco′s medium (IMDM) from Gibco Life Technologies (Gaithersburg, MD, USA), and ^3^H-thymidine from PerkinElmer (Waltham, MA, USA). A specification of polyclonal and monoclonal antibodies (mAb) used in this study is shown in Table 2.

**Table 1:** Specification of drugs used in this study

Name	Synonym	Supplier	Main target(s)
Bosutinib	SKI-606	ChemieTek	ABL, SRC
Dasatinib	BMS-354825	ChemieTek	ABL, SRC, KIT, BTK, LYN
Erlotinib	OSI-774	ChemieTek	EGFR, Her2
Gefitinib	ZD1389	ChemieTek	EGFR, Her2
Imatinib	STI-571	ChemieTek	ABL, KIT, PDGFR
Lapatinib	GW572016	ChemieTek	EGFR, Her2
Masitinib	AB1010	LC Laboratories	KIT, PDGFR, LYN
Midostaurin	PKC412	LC Laboratories	FLT3, KIT
Nilotinib	AMN107	ChemieTek	ABL, KIT, PDGFR
Sorafenib	BAY 43-9006	ChemieTek	Raf, VEGFR, KIT
Sunitinib	SU11248	ChemieTek	PDGFR, VEGFR, KIT
Everolimus	RAD001	ChemieTek	mTOR
NVP-BEZ235	BEZ235	ChemieTek	mTOR, PI3 kinase
Tozasertib	VX-680, MK-0457	ChemieTek	Aurora Kinase A, KIT
Vorinostat	SAHA	ChemieTek	HDAC

BTK, Bruton's tyrosine kinase; EGFR, epidermal growth factor receptor; PDGFR, platelet-derived growth factor receptor; VEGFR, vascular endothelial growth factor receptor; mTOR, mammalian target of rapamycin; PI3 kinase, phosphoinositide 3 kinase; HDAC, histone deacetylase.

**Table 2:** Specification of antibodies used in this study

Method	CD #	Name	Company	Clone	Isotype	Fluorochrome
FC	2	T11, LFA-2	BD-Pharmingen	RPA-2.10	mIgG1	PE
FC	9	BA2	Immunotech	ALB 6	mIgG1	FITC
FC	25	IL-2RA	BD-Pharmingen	2A3	mIgG1	PE
FC	30	TNFRSF8	BD-Pharmingen	BerH8	mIgG1	PE
FC	31	PECAM-1	Miltenyi Biotec	AC128	mIgG1	APC
FC	44	CDW44	BD-Pharmingen	515	mIgG1	PE
FC	54	ICAM-1	Immunotech	84H10	mIgG1	FITC
FC	58	LFA3	Immunotech	AICD58	mIgG2a	FITC
FC	62E	Selectin E	BD-Pharmingen	68-5H11	mIgG1	PE
FC	62L	Selectin L	BD-Pharmingen	DREG-56	mIgG1	PE
FC	62P	Selectin P	BD-Pharmingen	AK-4	mIgG1	PE
FC	63	LAMP-3	Immunotech	CLB-gran12	mIgG1	PE
FC	117	Kit	BD-Pharmingen	104D2D1	mIgG1	PE
FC	162	Selectin P ligand	BD-Pharmingen	KPL-1	mIgG1	PE
FC	–	Isotype	BD-Pharmingen	MOPC-21	mIgG1	FITC
FC	–	Isotype	BD-Pharmingen	MOPC-21	mIgG1	PE
FC	–	Isotype	BD-Pharmingen	X39	mIgG2a	FITC
ICC	2	T11, LFA-2	Novocastra	AB75	IgG1	n.a.
ICC	25	IL-2RA	Novocastra	4C9	IgG2b	n.a.
ICC	34	–	Novocastra	QBEnd/10	IgG1	n.a.
ICC	177	Kit	Dako	Polyclonal	n.a.	n.a.
ICC	–	HDC	Progen	Polyclonal	n.a.	n.a.
ICC	–	Tryptase	Dako	AA1	IgG1	n.a.
ICC	–	Chymase	Chemicon	B7	IgG1	n.a.

FC, flow cytometry; ICC, immunocytochemistry; FITC, fluorescein isothiocyanate; PE, phycoerythrin; APC, allophycocyanin; n.a., not applicable; HDC, histidine decarboxylase.

### Establishment of the NI-1 cell line

Mast cells were obtained from the peripheral blood of a 3.5-year-old mixed breed dog. At diagnosis, the patient presented with weight loss, splenomegaly, and lymphadenopathy. Based on blood and bone marrow examinations, the diagnosis of MCL was established. Despite splenectomy, the subject died because of acute hemorrhage from esophagus ulceration. Isolated MC were cultured in RPMI 1640 medium containing 10% FCS and antibiotics at 5% CO_2_ and 37°C. MC were passaged serially for more than 1 year. Then, cells were single-cell-cloned by limiting dilution. One clone, designated NI-1, was characterized in detail.

### Culture of C2 cells and HMC-1 cells

The canine mastocytoma cell line C2 (21) was kindly provided by Dr W. Gold (University of California, San Francisco, CA, USA). The human MCL line HMC-1 (22) was kindly provided by Dr J. H. Butterfield (Mayo Clinic, Rochester, MN, USA). Two subclones were used: HMC-1.1 harboring Kit V560G but not Kit D816V and HMC-1.2 harboring both mutants (6). C2 cells and HMC-1 cells were cultured in IMDM with 10% FCS and antibiotics at 5% CO_2_ and 37°C. Cells were passaged every 2–3 days and rethawed from an original stock every 6–8 weeks.

### Morphology and phenotyping

The morphology of NI-1 cells was evaluated by Wright–Giemsa staining. Immunocytochemistry (ICC) was performed as described (23) using antibodies depicted in Table 2. Flow cytometry was performed according to an established protocol (23) using fluorochrome-labeled mAb (Table 2). IgE receptor expression was analyzed by staining with fluorescein isothiocyanate (FITC)-labeled anti-IgE antibody A40-125F after preloading cells with IgE (Bethyl Laboratories, Montgomery, TX, USA). Antibody reactivity was determined on a FACSCalibur [Becton Dickinson (BD) Biosciences, San Jose, CA, USA]. Electron microscopy was performed essentially as described (24). In brief, NI-1 cells were washed and fixed in OsO_4_ for 45 min. Then, cells were again washed and incubated with uranyl acetate (5%). After dehydration, cells were embedded in resin and propylene oxide. Embedded cells were cut on an EM UC7 ultramicrotome (Leica, Wetzlar, Germany), transferred to copper grids, and then exposed to uranyl acetate and lead citrate. Sections were photographed using a JEM-1010 transmission electron microscope (JEOL Ltd, Tokyo, Japan).

### Sequencing of *Kit*

The original tumor samples as well as NI-1 cells (before and after xenografting) were examined for *Kit* mutations by sequencing analysis as described (14, 25). Three large fragments of the *Kit* cDNA product were amplified, gel-purified using the Qiaex II gel purification kit (Qiagen, Valencia, CA, USA), and sequenced through an automated sequencing technique using fluorescence-labeled dideoxynucleotides with capillary electrophoresis and an ABI sequence analyzer (Applied Biosystems, Foster City, CA, USA).

### Western blot experiments

Western blot experiments were performed essentially as described (6, 16) using antibodies against total Kit (Santa Cruz, Santa Cruz, CA, USA) and phosphorylated Kit (Cell Signaling Technology, Danvers, MA, USA). NI-1 cells, HMC-1.2 cells, and cord blood–derived cultured normal MC, generated as reported (26, 27), were examined by Western blotting. Cell lysates were separated in 7.5% SDS polyacrylamide gel electrophoresis, and antibody reactivity was made visible by donkey anti-rabbit IgG and Lumingen PS-3 detection reagent (all from GE Healthcare, Buckinghamshire, UK).

### Evaluation of effects of various TKI and other drugs on proliferation of MC

Cells were seeded in 96-well plates (10^4^ cells/well) and incubated with various targeted drugs (37°C, 48 h). In a first screen, drugs were applied at 0.1, 0.5, 1.0, and 2.0 μM. Effective drugs were then examined using additional concentrations. After incubation, 0.5 μCi of ^3^H-thymidine was added, and thymidine uptake was measured as reported (6). All experiments were performed in triplicate.

### Evaluation of drug-induced apoptosis in neoplastic MC

To quantify the expression of activated caspase-3 after drug exposure, flow cytometry was performed using an antibody against active caspase-3 (BD Biosciences). Before being stained, HMC-1 cells, C2 cells, and NI-1 cells were cultured in the presence or absence of targeted drugs (37°C; 24 or 48 h). Prior to staining, cells were fixed in formaldehyde (2%) and permeabilized using methanol (100%) at −20°C for 30 min. Expression of active caspase-3 was analyzed on a FACSCalibur (BD Biosciences). To confirm apoptosis in drug-exposed cells, a TUNEL assay was performed using the ‘In situ cell death detection kit-fluorescein’ (Roche, Mannheim, Germany) following the manufacturer's instructions.

### Evaluation of IgE-dependent histamine release

Histamine release experiments were performed in NI-1 cells and C2 cells using goat anti-dog anti-IgE antibody A40-125A (0.1–10 μg/ml) (Bethyl Laboratories) or Ca-ionophore A23187 (10 μg/ml) (Sigma-Aldrich, St. Louis, MO). Before being challenged with anti-IgE (37°C, 30 min), cells (1 × 10^5^/ml) were preloaded with dog IgE (Bethyl Laboratories) at 37°C for 2 h. In a separate set of experiments, IgE-preloaded NI-1 cells were incubated in control medium or medium containing masitinib (10 μM), imatinib (10 μM), or midostaurin (0.01–10 μM) at 37°C for 30 min before being exposed to anti-IgE (10 μg/ml) in histamine release buffer (Immunotech, Marseille, France). After incubation, cells were centrifuged at 4°C. Cell-free supernatants were examined for histamine content by radioimmunoassay (Immunotech) (27). Histamine release was expressed as percent of total histamine.

### Transplantation of NI-1 cells into NOD/SCID IL-2Rgamma^null^ (NSG) mice

Six-week-old male NSG mice (NOD.Cg-*Prkdc*^*scid*^ *Il2rg*^*tm1Wjl*^/SzJ) were purchased from Jackson Laboratory (Bar Habor, ME, USA). The study was approved by the ethics committee of the University of Veterinary Medicine Vienna and performed in accordance with guidelines for animal care and protection and protocols approved by the Austrian law (BMWF-66.009/0284-II/10b/2008). NI-1 cells or C2 cells (5 × 10^6^ cells in 0.2 ml) were injected subcutaneously into NSG mice (*n* = 5). Mice were monitored daily until mice developed palpable tumors. After resection, tumor samples were either frozen in liquid nitrogen or fixed in 10% neutral buffered formalin and embedded in paraffin. In addition, tumor nodules were cut into small pieces and digested in collagenase type II (Worthington Biochemical Corp, Lakewood, NJ, USA) to obtain single-cell suspensions as reported (24, 26).

### Statistical evaluation

To determine the significances in differences in proliferation and apoptosis in cells exposed to various drugs, the Student's *t*-test for dependent samples was applied. Results were considered statistically significant when *P* was <0.05.

## Results

### Morphologic and phenotypic characterization of NI-1 cells

In Wright–Giemsa-stained slides, NI-1 cells were found to be immature myeloid cells containing a round nucleus with fine chromatin and one or more nucleoli. The cytoplasm showed numerous vacuoles and a few metachromatic granules (Figure 1A). Electron microscopy confirmed that NI-1 cells are MC progenitors (Figure 1B). As assessed by ICC, NI-1 cells expressed tryptase (Figure 1C, Table 3) and Kit (CD117) (Table 3) as well as histidine decarboxylase (HDC), but did not express CD34 (Table 3). Flow cytometry experiments showed that NI-1 cells also express CD30 (Figure 1D). Expression of Kit was demonstrable in NI-1 cells by ICC (Table 3), flow cytometry (Figure 1E), and Western blotting (Figure 1G). In addition, NI-1 cells were found to express the IgER (Figure 1F). C2 cells were found to display a similar phenotype (Table 3). However, in contrast to NI-1, C2 cells expressed only low levels of IgER and CD30 (Table 3). As determined by radioimmunoassay (RIA), NI-1 cells contained 0.12 ± 0.06 pg histamine per cell, C2 cells 0.3 ± 0.17 pg per cell, HMC-1.1 cells 0.76 ± 0.46 pg per cell, and HMC-1.2 cells 0.4 ± 0.08 pg per cell (Table 3).

**Figure:**
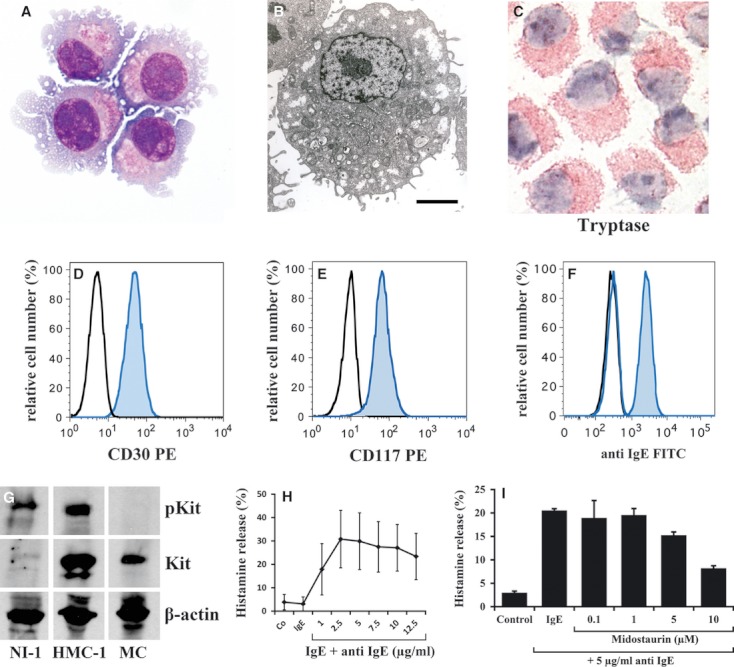
Phenotypic and functional characteristics of NI-1 cells. (A) NI-1 cells were stained by Wright–Giemsa method. As visible, NI-1 cells contained round nuclei with one or more nucleoli, numerous cytoplasmic vacuoles, and several cytoplasmic surface projections. (B) The ultrastructure of NI-1 cells was analyzed by electron microscopy using a JM-1010 transmission electron microscope as described in the text. Electron microscopy confirmed the presence of a mast cell (MC) progenitor, with numerous cytoplasmic surface projections, granule-like structures, and empty containers. Original magnification: ×2000, size bar represents 2 μm. (C) NI-1 cells were stained with an antibody against tryptase and analyzed by indirect immunocytochemistry as described in the text. (D, E) Expression of CD30 and CD117 on NI-1 cells was confirmed by flow cytometry using mAb against CD30 and CD117 (blue histograms). The isotype-matched control antibodies are also shown (black histograms). (F) NI-1 cells were pre-incubated with canine IgE at 4°C for 2 h, then with an FITC-labeled anti-canine anti-IgE antibody (blue histogram) for 30 min. The expression was analyzed by flow cytometry. The black histogram shows unstained NI-1 cells, and the blue open histogram represents NI-1 cells stained with the FITC-labeled anti-IgE antibody without IgE pre-incubation. Antibody reactivity was also controlled by an isotype-matched control antibody (not shown). (G) Western blot analysis of lysates of NI-1 cells, HMC-1.2 cells, and normal cord blood–derived cultured human MCs starved from SCF overnight. Western blotting was performed using an antibody against pKit and an antibody against total Kit. (H) NI-1 cells were preloaded with canine IgE and then incubated with various concentrations (as indicated) of anti-canine anti-IgE antibody at 37°C for 30 min as indicated. Thereafter, NI-1 cells were centrifuged at 4°C, and the supernatants and total suspensions were analyzed for histamine content by RIA. Histamine release results are expressed as percent of total histamine and represent the mean ± SD from four independent experiments. (I) NI-1 cells were pre-incubated with IgE for 2 h and then were washed and incubated with control medium or various concentrations of midostaurin (0.1–10 μM) at 37°C for 30 min, followed by challenge with anti-IgE antibody A40-125 (5 μg/ml) for another 30 min. Then, cells were centrifuged, and histamine content was determined in cell lysates and cell-free supernatants. Histamine release is expressed as percentage of total histamine and represents the mean ± SD of triplicates.

**Table 3:** Characteristics of cell lines

	NI-1	C2	HMC-1.1	HMC-1.2	NI-1 xeno-TX*
Diameter, size (μM)	11.2 (±0.7)	14.3 (±0.2)	17.7 (±0.7)	14.5 (±0.4)	12 (±0.6)
Histamine (pg/cell)	0.12 (±0.06)	0.3 (±0.17)	0.76 (±0.46)	0.4 (±0.08)	n.d.
*Kit* mutation status	2 SiM (Exon 8)	48-bp ITD (Exon 11)	V560G (Exon 11)	V560G (Exon 11)	2 SiM (Exon 8)
	MiM (Exon 8)			D816V (Exon 17)	2 MiM (Exon 8)
	12-bp dup (Exon 8)				12-bp del (Exon 10)
Immunocytochemistry	NI-1	C2	HMC-1.1	HMC-1.2	NI-1 xeno-TX*
CD2	(+)	(+)	(+)	(+)	(+)
CD25	+	+	+	+	n.t.
CD34	−	−	−	−	−
CD117/Kit	+++	+++	+++	+++	+++
HDC	+	+	++	+	n.t.
Chymase	−	−	−	−	−
Tryptase	+	++	+++	+++	+
Flow cytometry	NI-1	C2	HMC-1.1	HMC-1.2	NI-1 xeno-TX*
CD2	−	−	−	+++	−
CD9	+/−	−	+++	+++	−
CD25	−	−	−	+	−
CD30	+++	++	n.t.	n.t.	n.t.
CD44	+++	+++	+++	+++	+++
CD54	+	−	+	+++	+
CD58	−	+/−	++	+++	−
CD62E	+	−	+	+	+
CD62L	−	−	−	−	−
CD62P	−	−	−	−	−
CD63	−	−	+	+++	−
CD117/Kit	+++	+++	+++	+++	+++
CD162	−	−	+++	+++	−

SiM, single point mutation; HDC, histidine decarboxylase; MiM, missense mutation; bp, base pair; dup, duplication; del, deletion; ITD, internal tandem duplication; n.t., not tested; 0–10%: −; 11–20%: +/−; 21–45%: +; 46–70%: ++; 71–100%: +++.

The histamine content in cell lines was determined by RIA. Immunocytochemistry and flow cytometry were performed using anti-leukocyte antibodies as described in the text.

* NI-1 xenoTX: NI-1 cells obtained from mast cell tumors developing in NSG mice after xenotransplantation.

### NI-1 cells express a functional IgER

As visible in Figure 1H, incubation of IgE-preloaded NI-1 cells with anti-IgE resulted in a dose-dependent release of histamine, suggesting that NI-1 cells express a functional IgER. The Ca-ionophore A23187 also induced histamine liberation in NI-1 cells (data not shown).

### NI-1 cells form tumor lesions in NSG mice

To study the tumorigenicity of NI-1 cells, we injected these cells into the skin of NSG mice. Within 3 weeks, NSG mice were found to develop tumor nodules containing neoplastic MC. After resection and isolation, tumor-derived MC could again be cultured and were found to exhibit the same morphology, phenotype, and *Kit* mutations, compared to the original NI-1 clone (Table 3).

### NI-1 cells exhibit a unique profile of *Kit* mutations

As determined by Western blotting, NI-1 cells contained a constitutively active Kit receptor (Figure 1G). When examining the *Kit* mutation status, we found that NI-1 cells contain two silent mutations at nucleotide 414 and 507, and two missense mutation sites at nucleotide 107 (C to T) [amino acid 36 (P to L)] as well as at nucleotide 1187 (A to G) [amino acid 396 (Q to R)]. Furthermore, we found a 12-bp duplication mutation at nucleotide 1263 (repeat of AATCCTGACTCA) [amino acid 421–425 (insertion of amino acid QILT)] and a 12-bp shorter isoform of *Kit* at nucleotide 1550 (deletion of GTAACAGCAAG) [amino acid 517–520 (deletion of amino acid GNSK)]. The primary tumor lesion contained the same *Kit* mutations as that found in NI-1 cells. C2 cells were also examined for *Kit* mutations to confirm their identity. As expected, we were able to detect the 48-bp *Kit* internal tandem duplication (ITD) reported to be expressed in C2 cells (28).

### Effects of various antineoplastic drugs on proliferation of NI-1 cells

As assessed by ^3^H-thymidine uptake, a number of antineoplastic drugs were found to inhibit the proliferation of NI-1 cells (Figure 2A, Table 4). Among these drugs were the Kit kinase blockers midostaurin, dasatinib, sunitinib, tozasertib, imatinib, and sorafenib as well as drugs targeting Kit-downstream signaling molecules, including the mTOR blocker RAD001 and the PI3-kinase/mTOR-targeting drug NVP-BEZ235 (IC_50_ 0.01–0.5 μM). No major growth-inhibitory effects were seen with bosutinib, erlotinib, gefitinib, masitinib, and lapatinib (IC_50_ > 2 μM) (Table 4). However, the HDAC inhibitor vorinostat was found to block the growth of NI-1 cells (0.1–0.5 μM) (Table 4). The effects of the targeted drugs on caspase 3 activation, and thus their apoptosis-inducing effects on NI-1 cells and other MC lines, are shown in Table 5.

**Figure:**
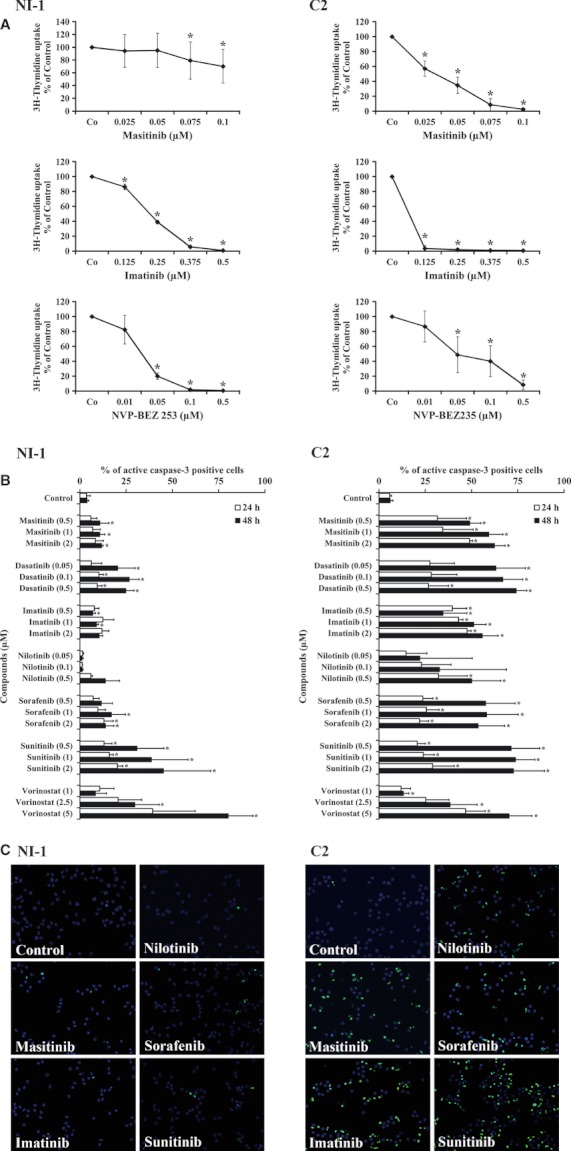
Effects of various drugs on proliferation and apoptosis of NI-1 cells and C2 cells. (A) NI-1 cells (left panels) and C2 cells (right panels) were incubated in control medium or with various concentrations of targeted drugs (masitinib, imatinib, NVP-BEZ235, as indicated) at 37°C for 48 h. Then, ^3^H-thymidine uptake was measured as described in the text. Results show the percent of control and represent the mean ± SD of at least three independent experiments. Asterisk: *P* < 0.05. (B) NI-1 cells and C2 cells were incubated in control medium or in medium containing various concentrations of targeted drugs (as indicated) at 37°C for 24 h (open bars) or 48 h (black bars). Then, cells were recovered, and the expression of activated caspase-3 was assessed by flow cytometry. Results show the percentage of active caspase-3-positive cells and represent the mean ± SD of three independent experiments. Asterisk: *P* < 0.05. (C) TUNEL assay experiments using NI-1 cells, C2 cells, and various targeted drugs. Cells were incubated in control medium or in medium containing masitinib (2 μM), imatinib (2 μM), nilotinib (2 μM), sorafenib (2 μM), and sunitinib (2 μM) as indicated (37°C, 24 h). The TUNEL assay was performed as described in the text. As visible, the targeted drugs applied induced apoptosis in C2 cells but not in NI-1 cells, confirming results obtained by flow cytometry with an antibody against active caspase-3.

**Table 4:** Effects of various drugs on proliferation of mast cell lines

	IC_50_ (μM)			
	
	NI-1	C2	HMC-1.1	HMC-1.2
Bosutinib	>2	>2	>2	0.5–1
Dasatinib	<0.003	0.003–0.006	0.0015–0.003	1–2
Erlotinib	>2	>2	>2	>2
Everolimus	<0.1	>2	>2	>2
Gefitinib	>2	>2	>2	>2
Imatinib	0.125–0.25	0.025–0.05	0.01–0.025	>2
Lapatinib	>2	>2	>2	>2
Masitinib	0.1–0.2	0.025–0.05	0.005–0.01	>2
Midostaurin	<0.1	<0.1	0.1–0.25	0.1–0.25
Nilotinib	0.2–0.3	0.1–0.2	0.025–0.05	1–2
NVP-BEZ235	0.01–0.05	0.01–0.05	0.05–0.075	0.01–0.05
Sunitinib	0.006–0.013	0.006–0.013	0.003–0.006	>2
Sorafenib	0.1–0.25	0.005–0.1	0.01–0.015	>2
Tozasertib	<0.1	>2	>2	<0.1
Vorinostat	0.1–0.5	0.1–0.5	0.5–1	0.5–1

Cells were incubated with various concentrations of targeted drugs at 37°C for 48 h. Then, ^3^H-thymidine uptake was measured. The mean IC_50_ values from at least three independent experiments are shown.

**Table 5:** Effects of targeted drugs on caspase 3 activation in neoplastic mast cells

	ED_50_ (μM)			
	
	NI-1	C2	HMC-1.1	HMC-1.2
Bosutinib	>5	>5	>5	>5
Dasatinib	>0.5	<0.05	<0.05	>2
Erlotinib	>5	>5	>5	>5
Everolimus	>5	>5	>5	>5
Gefitinib	>2	>2	>2	>2
Imatinib	>2	0.5–1	0.01–0.05	>2
Lapatinib	>2	>2	>2	>2
Masitinib	>2	0.5–1	0.05–0.1	>5
Midostaurin	>1	0.5–1	0.25–0.5	0.5–1
Nilotinib	>0.5	0.1–0.5	<0.05	>2
NVP-BEZ235	>5	>5	0.05–0.1	>5
Sunitinib	>2	<0.5	<0.05	>5
Sorafenib	>2	<0.5	0.05–0.1	>5
Tozasertib	>2	>2	2.5–5	>5
Vorinostat	2.5–5	2.5–5	2.5–5	>5

Cells were incubated with various concentrations of targeted drugs at 37°C for 48 h. Then, the percentage of caspase-3^+^ cells was determined by flow cytometry. The mean ED_50_ values from three independent experiments are shown.

### Effects of targeted drugs on other MC lines and comparison to NI-1 cells

C2 cells were found to respond clearly better to certain Kit kinase inhibitors (imatinib, masitinib) than NI-1 cells (Figure 2A). However, other kinase inhibitors including the PI3-kinase blocker NVP-BEZ235 and the mTOR blocker everolimus showed an even more potent antiproliferative effect on NI-1 cells compared to effects produced in C2 cells (Figure 2A, Table 4). We also compared drug responses in HMC-1 cells. As expected, HMC-1.1 cells were found to be more sensitive against several Kit TKI including imatinib, nilotinib, and masitinib than HMC-1.2 cells (Table 4). In addition, we found that sorafenib and sunitinib are potent inhibitors of growth of HMC-1.1 cells, whereas these TKI did not inhibit the growth of HMC-1.2 cells at pharmacologically meaningful drug concentrations (Table 4). All in all, the drug-response profile of NI-1 resembles more that of HMC-1.2 cells than that of HMC-1.1 cells, whereas the resistance profile of C2 resembles that of HMC-1.1 cells.

### Effects of various antineoplastic drugs on apoptosis in NI-1 cells and C2 cells

As assessed by flow cytometry, only sunitinib and vorinostat produced substantial caspase 3 cleavage in NI-1 cells (Figure 2B, Table 5). Less pronounced effects on caspase cleavage were seen with bosutinib and dasatinib. The other drugs tested, including masitinib (up to 2 μM) and imatinib (up to 2 μM) (Figure 2B, Table 5) as well as NVP-BEZ235 (up to 5 μM) and everolimus (up to 5 μM) (Table 5), did not induce apoptosis in these cells. By contrast, in C2 cells, various targeted drugs including masitinib, imatinib, nilotinib, dasatinib, sunitinib, sorafenib, and vorinostat were found to induce caspase cleavage at relatively low concentrations (Figure 2B, Tables 4 and 5). Apoptosis-inducing drug effects on NI-1 and C2 cells were confirmed by TUNEL assay (Figure 2C). We also compared responses to targeted drugs in HMC-1.1 and HMC-1.2 cells. Confirming previous data, HMC-1.1 cells were found to respond better to imatinib, nilotinib, dasatinib, and masitinib compared to HMC-1.2 cells (Table 5). In addition, we found that HMC-1.1 cells are more sensitive against sorafenib, tozasertib, and sunitinib compared to HMC-1.2 cells (Table 5). The other drugs tested showed comparable apoptosis-inducing effects on HMC-1.1 and HMC-1.2 cells (Table 5).

### Effects of various TKI on IgE-dependent histamine release in NI-1 cells

Patients with mastocytosis often suffer IgE-dependent mediator-related symptoms. We have recently shown that midostaurin blocks IgE-dependent histamine release in human MC. In the present study, midostaurin was found to inhibit IgE-mediated secretion of histamine in NI-1 cells in a dose-dependent manner (Figure 1I), whereas no effects were seen with masitinib or imatinib (not shown).

## Discussion

Mastocytomas are among the most frequent life-threatening neoplasms in dogs (29). During the past few years, Kit has been identified as a major molecular target in neoplastic MC in humans and canines (6–12). In both species, Kit-targeting TKI have been used to counteract the growth of neoplastic MC (6–12, 16–18). However, resistances against TKI have been described (10, 12, 18). Although the exact mechanisms remain unknown, several observations suggest that mutations in *Kit* and other genes may be involved. To better define the mechanisms of drug resistance in canine MC disorders, it seems important to create novel robust cell line models. We have established a novel canine MC line, NI-1, which harbors several *Kit* mutations and exhibits relative resistance against masitinib and other Kit-targeting TKI.

NI-1 cells were generated from a canine patient suffering from MCL. The origin of NI-1 from a MC progenitor was confirmed by morphologic studies, histamine content, electron microscopy, and immunophenotyping. An intriguing observation was that NI-1 cells express CD30, a marker antigen that is preferentially expressed in advanced mastocytosis (30). In line with this observation, NI-1 cells were found to form mastocytoma lesions in immunodeficient mice. Compared to C2 cells, NI-1 cells appeared to be a more immature and more rapidly growing tumor cell line *in vitro* and *in vivo*, which may be explained by the fact that NI-1 cells were generated from an extremely immature MC tumor.

Only a few well-characterized canine mastocytoma cell lines have been described so far (20, 31–33). In some of these cell lines, Kit-activating mutations have been identified. C2 cells express a Kit-activating ITD in exon 11 (29). However, this mutation does not confer resistance against imatinib or masitinib (16, 17). In NI-1 cells, several other *Kit* mutations, including missense mutations at nucleotides 107(C→T) and 1187(A→G), a 12-bp deletion at nucleotide 1550, and a 12-bp duplication at nucleotide 1263, were detectable. The latter mutation has been described to be a Kit-activating defect (15). Correspondingly, NI-1 cells were found to express a constitutively activated (phosphorylated) Kit receptor. Whether the 12-bp deletion at nucleotide 1550 causes resistance against various TKI such as masitinib and imatinib remains unknown.

Resistance against masitinib and other similar TKI is an important issues in the management and therapy of advanced canine MC tumors (13–15, 18–20). In fact, these patients have a poor prognosis. We asked whether our new cell line model can be employed to screen for targeted drugs that can overcome resistance against masitinib in canine MC tumors. Indeed, we were able to identify several drugs that effectively block the growth of NI-1 cells. Among these drugs were the PI3-kinase/mTOR blocker NVP-BEZ235, everolimus, and the TKI dasatinib, midostaurin, and sunitinib. These drugs produced growth inhibition (thymidine uptake) in NI-1 cells with an IC_50_ below 0.1 μM. In case of NVP-BEZ235 and everolimus, NI-1 cells were found to be even more sensitive cells compared to C2 cells. One explanation for this result is that the PI3-kinase/mTOR pathway plays a particular role for malignant Kit-triggered growth of NI-1 cells. A similar role of the PI3-kinase/mTOR pathway has been proposed for human neoplastic MC expressing Kit D816V (34). In fact, it has been described that HMC-1.2 cells exhibiting Kit D816V are more sensitive against the growth-inhibitory effect of rapamycin compared to HMC-1.1 cells (34). In the present study, this observation could be confirmed using the mTOR blocker everolimus and the mTOR/PI3-kinase blocker NVP-BEZ235. Both drugs exhibited more potent effects on the growth of HMC-1.2 cells than on HMC-1.1 cells in our experiments.

We also examined the effects of the mTOR inhibitors and other targeted drugs on survival and apoptosis in NI-1 cells and other MC lines. As expected, most TKI exerting growth-inhibitory effects also produced major apoptosis-inducing effects. However, as expected, the mTOR blockers, which may primarily act on neoplastic MC via cell cycle inhibition, did not show major pro-apoptotic effects on NI-1 cells.

In a substantial number of patients with (advanced) SM, mediator-related symptoms occur and represent a clinical problem (35–40). In several of these cases, IgE-dependent reactions can be documented (35–40). It has also been described that Kit-dependent and IgER-dependent pathways may act in concert to provoke mediator release in MC (41). However, so far, no MC lines expressing a functional IgER as well as relevant *Kit* mutations are available (32, 33). NI-1 cells were found to express the IgER on their surface. In addition, we were able to show that cross-linking of the IgER on NI-1 cells is followed by histamine release. Finally, we were able to show that midostaurin inhibits IgE-dependent secretion of histamine in NI-1 cells, which is in line with the observation that this TKI also blocks IgE-mediated histamine release in human MC and basophils (27).

In summary, we have established a novel canine MC line that may serve as a valuable tool for studying various aspects of neoplastic MC in advanced canine MC tumors. Specifically, this novel cell line should assist in exploring the mechanisms of abnormal growth and drug resistance in canine MC tumors, IgE-dependent activation of neoplastic MC, and the effects of various novel targeted drugs.
